# Differences in Salivary Cytokinome and Pathogen Load Between Rheumatoid Arthritis and Other Rheumatic Disease Patients

**DOI:** 10.3390/ijms26010197

**Published:** 2024-12-29

**Authors:** Aleksandra Korzeniowska, Agnieszka Daca, Maria Szarecka, Małgorzata Bykowska, Jacek Witkowski, Ewa Bryl

**Affiliations:** 1 Department of Physiopathology, Faculty of Medicine, Medical University of Gdansk, 80-210 Gdansk, Poland; agnieszka.daca@gumed.edu.pl (A.D.); jawit@gumed.edu.pl (J.W.); 2 Pomeranian Rheumatology Center, 81-759 Sopot, Poland; m.szarecka65@gmail.com (M.S.); malgorzata_bykowska@wp.pl (M.B.); 3 Department of Embryology, Faculty of Medicine, Medical University of Gdansk, 80-210 Gdansk, Poland

**Keywords:** rheumatoid arthritis, *Porphyromonas gingivalis*, Epstein–Barr Virus, cytokines, pathogenesis

## Abstract

Rheumatoid arthritis (RA), an autoimmune disease with complex pathogenesis, is characterized by an immune imbalance reflected, e.g., in the disturbed cytokines’ profile. Various viruses and bacteria can cause the upregulation of pro-inflammatory cytokines influencing RA development. In particular, oral cavity dysbiosis, observed in multiple chronic diseases including periodontitis, may be linked to RA. The cytokine profile (IL-1β, IP-10, IL-29, GM-CSF, IFN-α2, IFN-β, TGF-β1, MPC-1, TNF-α, IFN-γ, IL-6, IL-10, IL-17A, IL-12p70, IL-2, and IL-4) of RA patients’ saliva was evaluated using flow cytometry and benchmarked with their levels in saliva of healthy controls and patients with other rheumatic diseases. The levels of IL-1β, IP-10, IL-2, and IL-4 were significantly elevated in RA patients’ saliva compared to other studied groups. To define the potential role of the most suspicious microbial agents (Epstein–Barr Virus (EBV), Cytomegalovirus, Parvovirus B19, *Porphyromonas gingivalis*, and *Segatella copri*) for RA pathogenesis, the amounts of their DNA in the saliva of patients with RA were assessed in all the groups mentioned above. The EBV and *P. gingivalis* DNA levels measured by qRT-PCR were significantly higher in RA patients’ saliva than in other groups, indicating either the important role of these agents in RA pathogenesis or the higher susceptibility of RA patients for those infectious factors. The comprehension of the association of specific cytokine profiles in RA and the occurrence of specific viral and/or bacterial infections can be a key to a better understanding of RA pathogenesis. These results illustrate the complexity of the immunological profile of RA, show the high diagnostic potential of saliva, and provide insight into how various infections can contribute to RA development.

## 1. Introduction

The exact nature of rheumatoid arthritis (RA) outset is not well known. The diagnosis of RA depends on the coexistence of clinical symptoms and the occurrence of specific biomarkers, including antibodies against cyclic citrullinated peptide antigen (anti-CCP) and rheumatoid factor (RF) [1]. The pathogenesis of this autoimmune disease involves various agents, including genetic and environmental factors. Among the most common factors are HLADRB1 polymorphism, female hormones (especially estrogen), diet, cigarette smoking, viruses, and bacteria [2,3,4]. RA is considered as a systemic disease; outside the joints, it also adversely affects the cardiovascular system, bone marrow, immune system, and salivary glands [5,6,7]. RA is characterized by dysfunctions in both innate and adaptive immune systems. Cells of the innate system like macrophages, neutrophils, and natural killer (NK) cells are involved in joint inflammation. On the other hand, antigen-presenting cells (APCs), which can also belong to both the innate and adaptive immune system, participate in inflammation and secrete and stimulate the release of pro-inflammatory cytokines like interleukin-6 (IL-6) and tumor necrosis factor-α (TNF-α) [8]. The impact of those cytokines on the development of RA is indisputable. Not surprisingly, the development of therapies involving monoclonal antibodies capable of blocking the signaling of IL-6 and TNF-α is considered a milestone in the treatment of severe RA [9]. Besides IL-6 and TNF-α, different cytokines including granulocyte-macrophage colony-stimulating factor (GM-CSF), interleukin-1β (IL-1β), interleukin-2 (IL-2), interleukin-17 (IL-17), interleukin-21 (IL-21), and interleukin-23 (IL-23) can play an important role in RA development and are potential factors that are also considered in current and future RA therapies [10]. According to DrugBank information from June 11, 2024, inhibitors of some of the mentioned cytokines (Anakinra (IL-1 receptor antagonist), Secukinumab, and Ixekizumab (antibodies against IL-17A)) are approved for RA treatment [11]. The production of cytokines can be induced through various factors also involving bacterial and viral antigens [12]. Among cytokines typical for an immune reaction against a pathogen, type I interferon (IFN), type II interferon (IFN-γ), IL-1β, interleukin-12 (IL-12), and TNF-α can be distinguished. Moreover, the role of these cytokines in autoimmunity initiation is well established [8].

Different pathogens’ participation in autoimmunity development has been an object of research for decades. Latent or active viral infections, as well as bacterial infections or the disruption of microbiota homeostasis, can participate in RA pathogenesis [13]. Viruses can stimulate the immune system through various mechanisms, among which the most common are molecular mimicry, bystander activation, and epitope spreading [14]. Molecular mimicry is a process that occurs due to structural similarity between foreign antigens and self-peptides. It results in the activation of autoreactive T and B cells, and the production of pro-inflammatory cytokines [15]. Epstein–Barr Virus (EBV), Cytomegalovirus (CMV), and Parvovirus B19 (PB19) are often cited as major viral risk factors for autoimmune disease development. The antibodies directed against their elements are commonly detected among patients with RA in significantly higher titers than in healthy people [14].

The potential role of bacterial infection participation in autoimmunity development is becoming a growing topic of research nowadays. Particular attention has begun to be paid to the potential role of oral microbiome dysbiosis in the development of RA. The oral microbiome consists of symbiotic, commensal, and pathogenic organisms residing in the oral cavity. In a healthy oral microbiome, the vast majority of them are Gram-positive aerobic bacteria. Disbalance influenced by changes in the oral environment can lead to periodontitis (PD) development [16]. In PD, the healthy microbiome living in periodontal pockets is replaced by the Gram-negative anaerobic bacteria. PD is mostly caused by so-called “red-complex” bacteria including *Porphyromonas gingivalis*, *Tannerella forsythia*, and *Treponema denticola* [17]. In the context of the development of RA, *P. gingivalis* provokes the greatest interest. This bacterium, due to peptidyl-arginine deaminase (PAD) activity, is capable of citrullinating the host’s peptides, including fibrin and α-enolase, which results in the production of different anti-CCP antibodies, like anti-cyclic citrullinated peptide and anti-citrullinated enolase peptide (anti-CEP). These two types of ACPA (anti-citrullinated protein antibodies) can be detected in both PD and RA patients [18,19].

*Segatella copri* (*S. copri*, formerly *Prevotella copri*) is a gut microbiota bacterium commonly found in high abundance in RA patients’ fecal samples. Its potential role in RA pathogenesis may be due to the stimulation of the T helper (Th) 17 cell population and induction of Th17-cell-related cytokine production (IL-6 and IL-23) [20]. Moreover, *S. copri* can be found in the oral cavity of RA patients, which indicates that this bacterium, which is not only limited to the gut, can play a role in RA outset and development [7,21].

There are multiple papers evaluating cytokine levels in RA patients and the association of chosen pathogens with this disease, although presented results differ significantly between the sources, making their interpretation difficult [22,23]. Moreover, most studies show results of comparisons between RA patients and healthy volunteers only, even though the group of rheumatic diseases is far more diverse. In the vast majority of these studies, serum, plasma, or synovial fluid are used as a material for immunological or microbiological assessment, and there are very few reports on the use of saliva for this purpose. The cytokines levels in serum and mononuclear cells in the same group of patients were already measured by Brzustewicz E. et al. in a previous study. This study proved differences in cytokine concentrations between patients with undifferentiated arthritis and healthy controls. Moreover, the immune status of the patients changed during treatment [24]. In our study, we aimed to compare the levels of a broad spectrum of cytokines in the saliva of RA patients, group of patients with other rheumatic diseases (ORD), and healthy volunteers using the flow cytometry technique. Moreover, we also checked the saliva samples of people from the abovementioned groups for amounts of DNA from EBV, CMV, Parvovirus B19, *P. gingivalis*, and *S. copri* using a quantitative real-time polymerase chain reaction (qRT-PCR).

## 2. Results

### 2.1. Levels of Anti-Viral Response Cytokines

Our study aimed to assess the possible differences in the concentrations of saliva cytokines between RA patients and patients with other rheumatic diseases. Additionally, the cytokines levels obtained for RA patients were also compared with those present in the saliva of healthy volunteers (HC).

**Figure 1:**
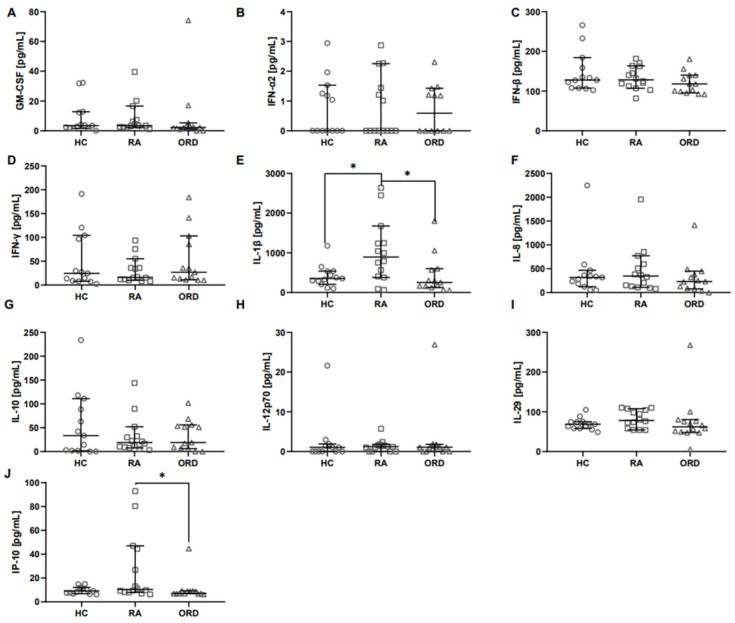
The differences in anti-viral response cytokine levels between RA patients and patients with other rheumatic diseases (ORD), as well as between RA patients and healthy controls (HC). Graphs show the concentrations of following cytokines: (**A**) GM-CSF, (**B**) IFN-α2, (**C**) IFN-β, (**D**) IFN-γ, (**E**) IL-1β, (**F**) IL-8, (**G**) IL-10, (**H**) IL-12p70, (**I**) IL-29, and (**J**) IP-10. Median with 95% Cl; Mann–Whitney U test, * *p* < 0.05.

Among all the analyzed cytokines from the anti-viral panel (GM-CSF, IFN-α2, IFN-β, IFN-γ, IL-1β, IL-8, IL-10, IL-12p70, IL-29, interferon gamma-induced protein 10 (IP-10)), only the concentrations of IL-1β and IP-10 were significantly higher in the saliva of RA patients than in the saliva of ORD patients (Figure 1B,E). Additionally, the IL-1β level was also significantly higher in RA patients’ saliva in comparison to healthy volunteers (Figure 1E).

### 2.2. Levels of Inflammation-Associated Cytokines

**Figure 2:**
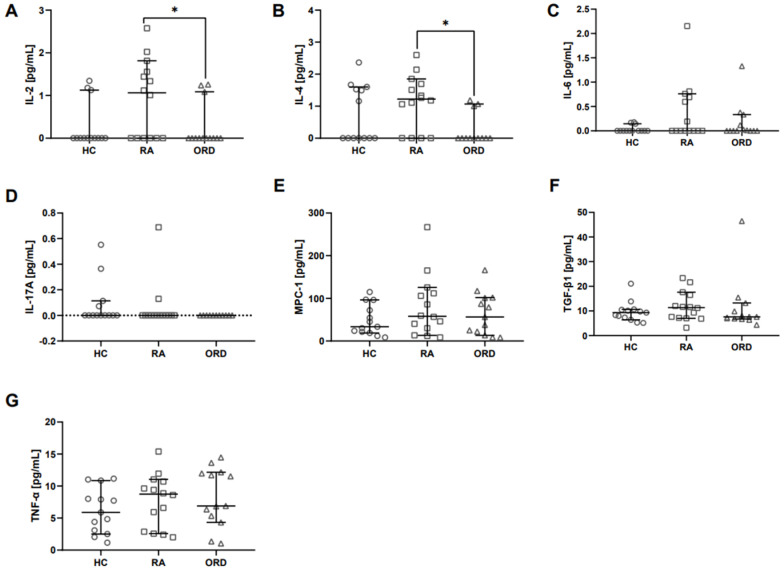
The differences in inflammation response cytokine levels between RA patients and patients with other rheumatic diseases (ORD), and the differences between RA patients and healthy controls (HC). Graphs show the concentrations of following cytokines: (**A**) IL-2, (**B**) IL-4, (**C**) IL-6, (**D**) IL-17A, (**E**) MPC-1, (**F**) TGF-β1, and (**G**) TNF-α. Median with 95% Cl; Mann–Whitney U test, * *p* < 0.05. The dotted line in graph (**D**) depicts the minimum value threshold equal to zero.

Surprisingly, out of the cytokines measured in the inflammation response panel, only the concentrations of non-inflammatory IL-2 and IL-4 were significantly higher in RA patients’ saliva in comparison to patients with ORD (Figure 2A and Figure 2B, respectively). The rest of the measured cytokines from that panel (IL-6, IL-17A, monocyte chemoattractant protein-1 (MPC-1), transforming growth factor β1 (TGF-β1), TNF-α) did not significantly differ between the studied groups (Figure 2C–G). There was also no significant difference between all measured cytokines in the saliva of RA patients and healthy subjects. Interestingly, much fewer saliva samples from RA patients contained undetectable levels of either IL-2 or IL-4 (6/14 for IL-2 and 4/14 for IL-4) compared to ORD (10/13 and 10/12, respectively) and to HC (10/13 and 7/13, respectively).

### 2.3. Numbers of Copies of Viral and Bacterial DNA

Three viruses (CMV, EBV, and Parvovirus B19) and two bacteria (*S. copri* and *P. gingivalis*) were detected and measured in the saliva samples of patients and healthy volunteers by qRT-PCR.

The results in all viral qRT-PCRs were considered positive at levels above 10 copies (cp)/µL. EBV loads above this level were obtained in 42.86% of the samples of DNA isolated from RA patients’ saliva, 15.79% of the samples of DNA isolated from healthy subjects, and 11.76% of the samples of DNA isolated from patients with other rheumatic diseases. The differences in proportions of positive samples were not significant. In the case of CMV and Parvovirus B19, all obtained results in every analyzed group were below 10 cp/µL; therefore, they were treated as negative ones.

**Figure 3:**
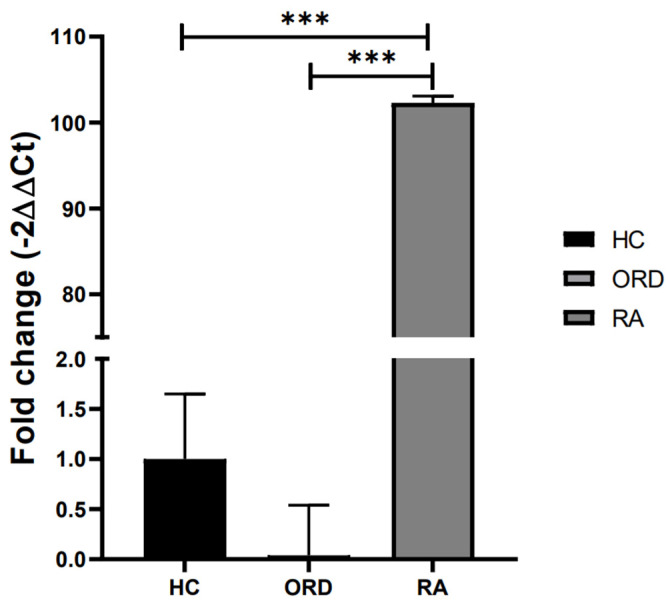
The differences in fold change (2^−ΔΔCT^) of EBV between RA patients, patients with other rheumatic diseases (ORD), and healthy controls (HC). Fold change with 95% Cl; Tukey’s multiple comparison test, *** *p* < 0.0001.

The comparison of fold change, obtained from a qRT-PCR by Tukey’s multiple comparison tests (Figure 3), revealed significantly higher EBV load in RA patients’ saliva samples in comparison to both healthy volunteers and patients with ORD. CMV and Parvovirus B19 DNA were not detected in all studied groups; therefore, no differences were present. In all cases, the internal control of each sample was at the expected level, ensuring the reliability of results.

In the case of *P. gingivalis*, positive samples were those with concentrations greater than 13 × 10^6^ cp/µL. *P. gingivalis* load above this level was obtained in 42.86% of the DNA saliva samples isolated from patients with RA, 5.88% of the DNA samples isolated from healthy subjects, and 18.75% of the DNA saliva samples isolated from patients with other rheumatic diseases. The only significant difference in the proportion of samples positive for *P. gingivalis* were between RA and HC (*p*-value = 0.0143). For *S. copri*, positive samples were obtained at a level higher than 10 cp/µL. *S. copri* load above this level was obtained in 7.69% of the DNA saliva samples isolated from RA patients, 23.52% of the DNA samples isolated from healthy subjects, and 10.52% of the DNA samples isolated from patients with other rheumatic diseases. In the case of *S. copri*, differences in proportions of positive samples were not significant.

**Figure 4:**
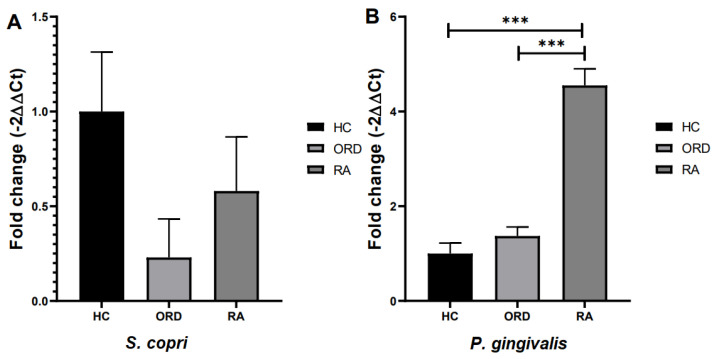
The differences in fold change (2^−ΔΔCT^) of selected bacteria between RA patients, patients with other rheumatic diseases (ORD), and healthy controls (HC). Fold change with 95% Cl; Tukey’s multiple comparison test, *** *p* < 0.0001. (**A**) Results for *S. copri*, (**B**) results for *P. gingivalis*.

Tukey’s multiple comparison test proved that the *P. gingivalis* fold change in DNA isolated from RA patients was significantly higher in comparison to both healthy subjects and patients with ORD (Figure 4A). There were no statistically significant differences in *S. copri* fold change (Figure 4B).

### 2.4. There Are Correlations Between Measured Cytokines and Patients’ Clinical Features

**Figure 5:**
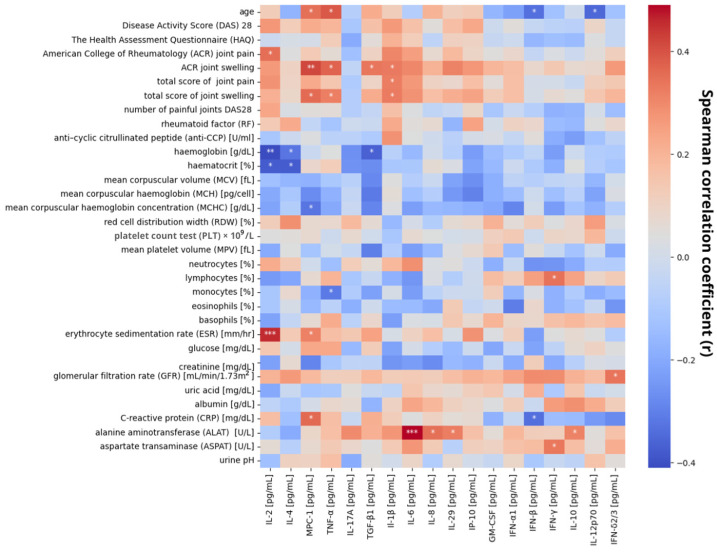
Heatmap of correlation of measured cytokine levels with patients’ clinical features (* *p* < 0.5, ** *p* < 0.01, *** *p* < 0.005 by Spearman’s correlation test).

Figure 5 presents results from the Spearman correlation analysis between clinical features of patients and cytokines levels in their saliva samples. At *p* < 0.01, statistical significance was observed for correlations between hemoglobin level and IL-2 level, as well as joint swelling in ACR scale and MPC-1 level, where the first correlation was negative and the second was positive. Moreover, with significance at *p* < 0.005, correlations between IL-2 level and erythrocyte sedimentation rate (ESR), IL-6 level and ALAT, and IL-29 level and the number of swelling joints in the DAS28 scale were observed. All of these correlations were positive. Other correlations with statistical significance with at least *p* < 0.05 are also presented in Figure 5.

**Figure 6:**
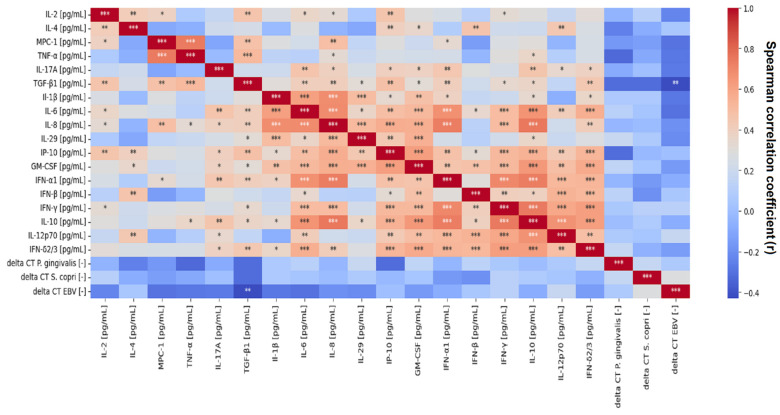
Heatmap of correlation of cytokine levels with delta Ct obtained from qRT-PCR (* *p* < 0.5, ** *p* < 0.01, *** *p* < 0.001 by Spearman‘s correlation test).

Figure 6 shows the correlations between concentrations of all measured cytokines and their delta Ct values obtained from qRT-PCR. To generate the plot, Spearman coefficients and *p*-values were computed. Statistically significant correlations were present in the abundance between cytokines, and most of them were positive ones. Delta CT results obtained from qRT-PCR in the vast majority did not correlate with cytokines and each other in a statistically significant way. The only exception was the value of Delta Ct obtained for EBV, which significantly (*p* < 0.05) correlated with the level of TGF-β1, lowering its concentration in saliva.

## 3. Discussion

In our study, we aimed to compare levels of cytokines, as well as viral and bacterial DNA levels, in the saliva of RA patients, patients with other rheumatic diseases, and healthy volunteers. For cytokine measurement, we chose flow cytometry, as this technique allows for the determination of many cytokines levels at once. To determine the amount of DNA in saliva, we used qRT-PCR, according to the ease, speed, and accuracy of this method. Both of these methods allowed us to limit the amount of material used for analysis. Because we aimed for saliva as our experimental material, the volume of accessible material was a crucial limiting factor, as it is well known that, in many rheumatic diseases, the problem with salivary gland activity is quite common.

One of the measured cytokines was IL-1β, a pro-inflammatory cytokine, commonly detected in higher levels in the sera of RA patients [25]. IL-1β is produced by monocytes and macrophages [26] as a precursor protein termed pro-IL-1β; the mature protein is created due to the activity of caspase-1 triggered in the NLRP inflammasome [27]. This cytokine plays a crucial role in RA pathogenesis and progression by inducing the production of other cytokines (e.g., chondrocytes and synovial mononuclear cells), enhancing matrix metalloproteinases (MMPs) release by fibroblasts, and consecutive bone erosion [28]. We observed significantly higher levels of IL-1β in the saliva of RA patients in comparison to healthy volunteers. Apart from the abovementioned impact of IL-1β on MMP activity, it is also one of the major inflammatory mediators, and an elevated level of this cytokine is commonly observed during chronic inflammatory diseases [29]. In another study, a higher level of IL-1β was also detected in samples from ORD patients (ankylosing spondylitis [30] and osteoarthritis [31]). Our observations indicated elevated levels of this cytokine in RA patients’ saliva in comparison to ORD, which can suggest the more extensive role of this cytokine in RA than in ORD; but, on the other hand, it can be the result of selecting different biological material (saliva in our study and sera in the cited papers) for analysis. Moreover, we found out that the level of IL-1β positively correlates with IP-10, the level of which was also significantly higher in RA patients in comparison to healthy subjects. This result may have been because both of these molecules are produced during ongoing inflammation, but there are no reports at this point suggesting, e.g., positive feedback between them or any other type of cooperation suggesting their possible net effect on RA development. IP-10 is a chemokine, the production of which is induced by IFN-γ in various cell types including macrophages, endothelial cells, and fibroblasts [32]. We found it not surprising, then, that there is a strong positive correlation between IFN-γ and IP-10 levels in RA patients’ saliva. Like other chemokines, IP-10 plays a role in leukocyte trafficking and can be involved in various infectious and inflammatory processes, as well as autoimmune rheumatic disease development [33]. According to Lee et al.’s studies, IP-10 induces cell migration through C-X-C chemokine receptor 3 (CXCR3) and can play a role in the progression of arthritis by CXCR3 and toll-like receptor-4 (TLR4) [32].

IL-2 is known for its immunomodulatory effect, mostly including the regulation of T cells. IL-2 plays an important role in the development and function of T regulatory cells and is also an important molecule in ensuring the proper function of conventional T cells [34]. According to our study, the IL-2 levels in RA patients’ saliva were significantly higher in comparison to ORDs’ and were detectable in the majority of patients’ saliva samples. Moreover, IL-2 correlates with such parameters as the ESR, level of hemoglobin [g/dL], hematocrit [%], and joint pain in ACR scale. All these parameters reflect disease activity, which indicates that the level of this cytokine is associated with disease severity. That is not surprising per se, because similar findings were already observed in Baochen Li et al.’s study of the RA patients’ sera, where elevated levels of IL-2 were associated with the disease activity parameters, such as ESR, DAS28, and C-reactive protein (CRP). The IL-2 level can impact the disease by influencing the balance between Th17 and Treg cells, where dysregulation is one of the key mechanisms in RA pathogenesis [35]. There is also a relationship between IL-2 level and natural killer (NK) cells, where IL-2 can enhance NK cell cytotoxicity. Interestingly, according to Kogure T et al.’s study, patients with RA are characterized by a lower induction of killer cell inhibitory receptors (KIRs) in response to IL-2. This can be a major reason for the higher susceptibility of RA patients to microbial infections [36].

The last out of the analyzed cytokines, whose level was significantly higher in RA than in ORD patients, was IL-4. This anti-inflammatory cytokine can be produced by various cells like mast cells, basophils, and eosinophils, but, in vast majority, it is produced by T cells. IL-4 is a member of the Th2 cytokine family, with IL-13 inducing the differentiation of naïve T cells into Th2 cells and the activation of B cells and production of antibodies [37]. Moreover, IL-4 plays a crucial role in the regulation of the cells’ proliferation and apoptosis [38]. It would seem logical that, because of its function in differentiating T cells into Th2 cells and the reduction in cytokine production by Th1 cells, increased levels of IL-4 would have the effect of reducing disease severity in patients with RA [39]. The treatment of a collagen-induced arthritis (CIA) mouse model with anti-IL-4 antibodies leads to increased severity of the disease, though, which is associated with the exacerbation of inflammatory reaction. This can indicate the protective role of IL-4 in RA [40]. Even though elevated levels of IL-4 are commonly found among RA patients in different stages of the disease [41,42], some studies suggest that IL-4 is necessary for the disease outset. In some cases, the reduction in IL-4 decreases the amount of autoantibodies, which correlates with diminished disease severity. Due to these observations, IL-4 can play both roles in RA, pathogenic and protective, and a better understanding of its role in RA is necessary [43].

Other studied cytokines, TGF-β1, MPC-1, TNF-α, IL-6, IL-8, IL-10, IL-17A, IL-12p70, IL-29, GM-CSF, IFN-α2, IFN-β, and IFN-γ, did not show any saliva concentration differences between all studied groups. Most of these cytokines play an important role in RA pathogenesis and progression. The usage of inhibitors to some of these cytokines, especially IL-6 and TNF-α in RA therapy, bring very good results in disease suppression [44]. Our results may be explained by the small number of patients in the analyzed groups and the use of saliva as a diagnostic material instead of serum or plasma. Some cytokines are produced directly in the mouth (IL-1β, IL-6, TNF-α), and their concentration in the mouth can be even higher than in the blood. On the other hand, some cytokines (IL-10, IFN-γ) are at lower concentrations in saliva than in blood [45]. The same conclusion can be drawn from our study, where some cytokines are at barely detectable concentrations or below the scale of quantification. Expanding study groups and/or changing the analytical method, for example, to quantitative PCR, which is proven to be more sensitive than flow cytometry, could potentially lead to statistically significant results. However, the detection of mRNA for cytokines will not exactly correlate with the amount of cytokine itself. In our opinion, we measured the possible working concentration of cytokines, which depends both on the amount of production and the possible turnover of the cytokines of interest.

Apart from the cytokines levels, we checked whether there were differences in the specific pathogen load in the saliva samples of RA, ORD, and HC individuals by measuring the amount of DNA from EBV, CMV, and Parvovirus B19 viruses as well as *P. gingivalis* and *S. copri* bacteria. Among all of the analyzed viruses, only the load of EBV was significantly higher in RA patients’ saliva than in ORD patients and healthy volunteers. All these viruses are known as possible risk factors for RA development and are typically found in the vast majority of society. Among them, EBV is the most common and it is present in almost 99% of the population [46]. The obtained results are probably not because of the active phase of primary infection because, in contrary to anti-viral antibodies (EBV nuclear antigen 1 (EBNA-1), IgG and capsid antigen (CA) IgG), which persist for life [46], the level of EBV DNA in saliva decreases with time [47]. That can indicate a possible role of viral reactivation in the outset and/or progress of RA. Interestingly, a higher level of EBV DNA in the saliva of RA patients is possibly due to more intensive replication of this virus in the oral epithelia of RA patients. Fetchner S. et al. observed the increased EBV reactivation cycles among RA patients in preclinical stages, which can support our hypothesis [48]. Nonetheless, this possibility should be independently confirmed through the detection of certain biomarkers of reactivation (EBNA-1, CA, and early antigen-diffuse (EA-D)). During replication, the virus expresses EBNA-1, a major EBV epitope, which was also detected at higher levels in RA patients. Molecular mimicry between some autoantigens and EBNA-1 can lead to tolerance breakdown and the initiation of the disease [49]. Moreover, a higher level of EBV DNA in saliva correlates negatively with TGF-β1 levels. The same situation is observed in EBV-related cancers. TGF-β1 plays a part in the modulation of cell proliferation, adhesion, differentiation, and survival. Moreover, it is a tumor suppressor, and abnormalities in its functioning can lead to the development of cancer and other diseases [50].

*S. copri* and *P. gingivalis* DNA levels in saliva were the last measured parameters in all studied groups. The level of *P. gingivalis* DNA was significantly elevated in saliva from RA patients; the *S. copri* level did not differ between the studied groups though. *S. copri* is a gut microbiota bacterium that is also commonly found in the salivary glands of RA patients [21]. The lack of differences between analyzed groups in our study is probably due to their small size. Our results on *P. gingivalis* were in line with those reported previously in the literature, confirming the association of *P. gingivalis* with the pathogenesis of RA [23]. Cytokines, produced in both RA and PD, play an important role in their pathogenesis. Some cytokines like IL-1β, IL-17A, IFN-γ, and IL-10 are elevated in gingival crevicular fluid from PD patients [51]. Surprisingly, we did not find any correlations between these cytokines and *P. gingivalis* level. An explanation of these observations can be that the studied group was small, but there was also the limitation of the material used. Especially interesting in the case of PD and RA connection is IL-17A, whose overproduction is mentioned as linked with chronic inflammation and autoimmune disease development. It is also responsible for the stimulation of releasing other cytokines connected with RA and PD, such as IL-6, IL-8, IL-1β, and IL-10 [52]. In this study, we also observed the correlations between the mentioned cytokines (except IL-1β) and IL-17A, which confirms the regulatory function of this cytokine.

### Limitations of the Study

The authors are aware of the limitations of this study. The first limitation is due to the small number of patients in all analyzed groups. Although RA is known to be the most common rheumatic disease, its prevalence is still around 1% in the general population, which makes patient recruitment much more difficult, especially considering that only patients before treatment were recruited. Moreover, the levels of some cytokines are at the limit of quantification and even below. This is due to a still-new concept of the determination of cytokine levels in saliva by flow cytometry, and a lower concentration of some cytokines in comparison to serum. Moreover, in the case of RA patients, the lower activity of the salivary glands can be observed. The manifestation of this can be seen in lower flow rate, reduced pH of saliva, and high incidence of oral dryness [53,54]. Rinderknecht C. et al. discovered that a high flow of saliva can reduce the amount of cytokines, but the direct impact of salivary gland activity on the immune composition of saliva is not well known [55]. Additionally, in our study group, patients with Sjogren syndrome were not included. Despite these limitations, we proved that saliva can be a worthwhile diagnostic material and, even though the analyzed populations of patients are small, there are still some differences between rheumatoid arthritis and other rheumatic diseases worth noticing. On the other hand, it is also important to remember that the usage of saliva as a diagnostic material instead of blood can generate some logistical and cost implications. The collection of saliva is non-invasive (special test tubes are used instead of needles) and does not require qualified personnel, which is needed in the case of blood collection. These things can lower the cost of material collection. Saliva is less stable than blood, though, and requires specific storage and transport procedures, which can also generate additional costs. Depending on the length of time the sample has to withstand until analysis, the cost of saliva usage can be higher or lower than blood. Nonetheless, the use of saliva can be the most practical for those patients who need to be under medical control and have a regular examination, but blood collection is limited due to some aspects, like anemia.

## 4. Materials and Methods

### 4.1. Clinical Sample Analysis

The saliva samples from 15 patients with diagnosed RA, 19 patients with other rheumatic diseases (see Table 1 for details), and 20 healthy age- and sex-matched controls (HC) were collected using Salivette^®^ tubes (51.1534, Sarstedt, Nümbrecht, Germany). The saliva was obtained from all recruited patients before starting any treatment. All tubes were centrifuged at 2250 rpm for 3 min; after that, samples were stored at −80 °C until further use. Patients participating in the study were enrolled at the Regional Hospital for Rheumatic Diseases in Sopot, Poland. Both patients and controls were subjected to a series of laboratory tests, including RA serological markers like RF and anti-CCP, as well as ESR and assessment of concentrations of CRP, as well as blood count, alanine aminotransferase (ALT), aspartate aminotransferase (AST), mean cell haemoglobin (MCH), and others. A clinical assessment of the patient’s condition was also conducted, which included disease activity score (DAS28), swollen joint count (SJC), tender joint count (TJC), American College of Rheumatology (ACR) tender score, ACR swollen score, and Health Assessment Questionnaire (HAQ). Laboratory and clinical data not included in Table 1 are attached in Appendix A and Appendix B.

This study was approved by the Local Independent Committee for Ethics in Scientific Research at the Medical University of Gdansk, and written consent was obtained from all patients and healthy volunteers (NKBBN/389/2012).

### 4.2. Cytokine Quantification by Flow Cytometer

Cytokine level determination in saliva was conducted using two LEGENDplexTM sets: LEGENDplexTM HU Essential Immune Response Panel w/VbP (740930, BioLegend, San Diego, CA, USA) and LEGENDplexTM Human Anti-Virus Response Panel w/VbP (740390, BioLegend, San Diego, CA, USA) according to the manufacturer’s protocol. These sets allow for the semiquantitative estimation of the concentrations of IL-1β, IP-10, IL-29, GM-CSF, IFN-α2, IFN-β, TGF-β1, MPC-1, TNF-α, IFN-γ, IL-6, IL-10, IL-17A, IL-12p70, IL-2, and IL-4.

Briefly, the detection beads were sonicated to remove aggregates and the Standard Cocktail was diluted to yield initial concentrations of measured cytokines and then serially diluted in the assay buffer to allow for the creation of the standard curve. Saliva and standard samples (25 µL) were separately mixed with 25 µL of the assay buffer and 25 µL of vortexed pre-mix of detection beads in a 96-well plate and agitated at 800 rpm/min for 2 h at room temperature. After incubation, the beads were centrifuged and washed once with the wash buffer and incubated for 1 h at RT with the Detection Antibodies. Next, 25 µL of streptavidin-phycoerythrin (SA-PE) was added to each well and incubated for half an hour prior to final washing and dilution of beads in 150 µL of wash buffer. The samples were then transferred to cytometric tubes and analyzed using the flow cytometer (BD FACSAriaTM III Cell Sorter, Becton, Dickinson and Company, Franklin Lakes, NJ, USA).

### 4.3. Cytometric Raw Data Analysis

Concentration of cytokines in each saliva sample and the standard were measured in duplicates; therefore, the mean value of obtained results was used in further statistical analysis. The raw cytometric data were analyzed using FlowJo 10.8.0 (Becton, Dickinson and Company, Franklin Lakes, NJ, USA). The mean fluorescence intensity (MFI) of phycoerythrin (PE) emitted by the Detection Antibody was used to measure the concentration of each analyte.

The cytokine concentrations were calculated with GraphPad Prism version 8.0.1 (GraphPad Software, San Diego, CA, USA). Concentrations were estimated using x = log(x) transformation and non-linear regression matching by the method of least squares and expressed as pg/mL.

### 4.4. Viral DNA Extraction

Viral DNA extraction was performed using a Viral DNA/RNA kit (034-100, A&A Biotechnology, Gdansk, Poland) according to the manufacturer’s protocol. Briefly, 100 µL of saliva, 400 µL R9F buffer, and 4 µL of universal internal control (UNIC/GP/050, GeneProof, Brno, Czech Republic) were mixed. After that, the mixture was vortexed and incubated for 10 min at room temperature. Next, 250 µL of isopropanol was added and mixed by inverting the sample several times. The entire volume was then applied to mini-columns from the kit and was centrifuged at 13,400 rpm for 1 min. The mini-columns were placed in new tubes and washed with 700 µL of A1 flush buffer. After centrifugation, the filtrate from the tubes was removed and 300 µL of A1 flush buffer was added and washed again. In the next step, the mini-columns were placed in sterile tubes and washed with 40 µL of nuclease-free water to elute DNA. Tubes were incubated at room temperature for 3 min and centrifuged at 13,400 rpm for 1 min. The DNA concentration was measured using a spectrophotometer (EpochTM, BioTek^®^ Instruments, Winooski, VT, USA). After that, the probes were stored at −20 °C until further determination. The same procedure was carried out with nuclease-free water, which was later used in qRT-PCR as a negative control.

### 4.5. Bacterial DNA Extraction

Bacterial DNA extraction was performed using a Genomic Mini AX Bacteria+ kit (060-60M, A&A Biotechnology), according to the manufacturer’s protocol. Briefly, 1 mL of saliva was suspended in 1 mL 2xBS (bacterial suspension) buffer and precisely mixed. To the mixture, 4 µL of lysozyme (50 mg/mL) and 10 µL of mutanolysine were added, mixed, and incubated at 50 °C for 40 min. After that, 2 mL of L1.4 lysing solution and 40 µL of proteinase K were added and incubated at 50 °C for 20 min. During the incubation, the solution was mixed by inverting tubes multiple times. In the meantime, to prepare columns, 800 µL of K1 balancing solution was applied. After the incubation was ended, the tubes were intensively vortexed for 15 s and centrifuged at 13,400 rpm for 5 min. The supernatant was then transferred to balanced columns for gravitational elution. After that, 1.5 mL of K2 rinse solution was added to the column and, again, gravitational elution followed. This step was repeated two times and, finally, 100 µL of K3 elution solution was added. Columns were transferred to precipitation tubes and 1 mL of K3 elution solution was added. In the next step, to precipitate the proteins, 800 µL of PM (precipitation mixture) was added, mixed, and centrifuged at 10,000 rpm for 10 min. The supernatant was carefully removed, and the pellet was resuspended in 500 µL of ethanol (70%), mixed, and centrifuged at 10,000 rpm for 3 min. Again, the supernatant was removed and the pellet was dried for 5 min at room temperature. After that, the pellet was resuspended in nuclease-free water (40 µL). The DNA concentration was measured spectrophotometrically directly after the extraction, and the material was stored at −20 °C until further use.

### 4.6. Real-Time Quantitative Polymerase Chain Reaction—Viral Assay

The detection and quantification of viral DNA in saliva was carried out with GeneProof diagnostic tests (GeneProof a.s., Brno-jih, Czech Republic), matched to the sought virus (GeneProof Parvovirus B19 PCR Kit (B19/ISEX/100), GeneProof Epstein–Barr Virus (EBV) PCR Kit (EBV/GP/100), and GeneProof Cytomegalovirus (CMV) PCR Kit (CMV/ISEX/100)). All used kits contained positive controls; moreover, internal controls were added to all samples. Briefly, 5 µL of a DNA sample containing internal control was mixed with 15 µL MasterMix and added directly into the wells of a 96-well PCR plate and used in qRT-PCR. Thermal cycling was performed according to the protocol in a PikoReal 96 Real-Time PCR System (Thermo Scientific, Waltham, MA, USA) with 1 cycle of PCR activation at 95 °C for 10 min, followed by 45 amplification cycles, each consisting of a denaturation step (95 °C, 5 s), annealing (60 °C, 40 s), and elongation (72 °C, 20 s). The fluorescence intensity was measured at FAM and HEX channels.

### 4.7. Real-Time Quantitative Polymerase Chain Reaction—Bacterial Assay

The qRT-PCR detection and quantification of *P. gingivalis* and *S. copri* in isolated DNA was performed with DyNAmo™ ColorFlash SYBR^®^ Green qPCR kit (F416L, Thermo Scientific, Waltham, MA, USA). Isolated DNA (at a final concentration of 10 ng/µL) was added to the mixture containing 10 µL of MasterMix (Thermo Scientific, Waltham, MA, USA) and 1 µL of primers specifically for bacteria (Table 2; Genomed S.A., Warsaw, Poland), 0.1 µL ROX reference dye, and nuclease-free water. The final reaction volume was 20 µL. The qRT-PCR was performed in 96-well PCR plates in a PikoReal 96 Real-Time PCR System. The thermal cycling included the following steps, obtained experimentally: activation at 95 °C for 3 min, followed by 40 cycles of denaturation at 95 °C for 15 s, annealing at 55 °C (*S. copri*) or 51 °C (*P. gingivalis*) for 30 s, and elongation on 72 °C for 30 s. Each sample was also prepared with starters for 16S rRNA considered as the internal control for bacterial DNA. The negative control was a sample where DNA was replaced by nuclease-free water, while the positive controls were *S. copri* DNA (DSM 18205, Leibniz Institute DSMZ-German Collection of Microorganisms and Cell Cultures GmbH, Braunschweig, Germany) in a concentration of 1 × 10^2^ copies/µL and *P. gingivalis* (DSM 20709, Leibniz Institute DSMZ-German Collection of Microorganisms and Cell Cultures GmbH, Braunschweig, Germany) in a concentration of 1 × 10^7^ copies/µL.

### 4.8. qRT-PCR Raw Data Analysis

The change fold obtained for each group for all sought bacteria and viruses was calculated with the 2(-delta delta C(T)) method (2^−ΔΔCT^).

The significance of differences in positive sample proportion among groups was evaluated using the Chi-square test in GraphPad 8.0.1 statistical software (GraphPad Software, San Diego, CA, USA), version 8.0.1.

### 4.9. Statistical Analysis

Shapiro–Wilk test was used to evaluate the normality of flow cytometric data distribution. Because the data were not normally distributed, non-parametric tests were applied for further analysis.

Mann–Whitney U test for two groups comparison (e.g., HC vs. RA) and Kruskal–Wallis test for three-group comparison (HC vs. RA vs. ORD) were used. The results with *p* < 0.05 were considered statistically significant.

Parametric Tukey’s multiple comparison test was used to interpret normalized data of 2^−ΔΔCT^ analysis of qRT-PCR results.

The potential correlations between patients’ clinical features and cytokine levels, as well as qRT-PCR results, were assessed using the Spearman correlation test. The results are presented in the heatmap, retrieved from Python version 3.11, including libraries like pandas, matplotlib, seaborn, and scipy stats.

## 5. Conclusions

Due to the complexity of RA pathogenesis, some mechanisms of its outset are still not known or ambiguous. Although viruses’ and bacteria’s role in RA development is almost certain, a better understanding of their role in RA pathogenesis is still needed, especially in the context of diagnosis and possible strategies of treatment. The vast majority of RA diagnoses are based on clinical features and two basic biomarkers: ACPA and RF. In this study, we determine that cytokines, viruses, and bacteria seem to play an important role in RA pathogenesis. Moreover, the levels of all measured factors can be detected in saliva, which, in the future, can limit the usage of blood for diagnosis. Our findings are another step toward a better understanding of the pathogenesis of RA, although many of the mechanisms and links in the process remain unknown.

## Figures and Tables

**Table 1:** Basic characteristics of patients with RA, ORD, and HC.

Patients	Sex	Age	Diagnosis	DAS-28	RF (UI/mL)	Anti-CCP (U/mL)	ESR (mm/h)	CRP (mg/dL)
ORD 1	female	42	other	1.81	<10		6	0
ORD 2	female	41	psoriatic arthritis	3.86	<10	<7	23	5.9
ORD 3	female	22	other	4.56	<10	<7	26	0.4
ORD 4	female	32	psoriatic arthritis	3.60	<10	<7	18	1.3
ORD 5	female	34	osteoarthritis	0.64	17	<7	2	0.3
ORD 6	female	38	other	2.10	<10	<7	2	0.3
ORD 7	female	24	other	1.68	<10	<7	2	0
ORD 8	female	46	other	2.91	<10	<7	4	0
ORD 9	female	43	osteoarthritis	1.36	<10	<7	4	0.5
ORD 10	female	23	ankylosing spondylitis	4.17	96	<7	3	0
ORD 11	female	20	ankylosing spondylitis	3.38	23	<7	2	0
ORD 12	female	46	osteoarthritis	2.04	<10	-	17	1.3
ORD 13	female	52	osteoarthritis	3.53	<10	<7	15	0
ORD 14	female	53	osteoarthritis	0	<10	<7	26	0.8
ORD 15	female	56	other	0	<10	<7	9	0.5
ORD 16	female	43	other	1.95	<10	-	-	-
ORD 17	female	56	psoriatic arthritis	4.90	<10	<7	8	2.8
ORD 18	female	44	psoriatic arthritis	2.02	<10	<7	7	0
ORD 19	male	36	ankylosing spondylitis	0.90	<10	<7	2	0.7
RA 1	female	44	rheumatoid arthritis	4.65	21	268.80	8	1.3
RA 2	female	61	rheumatoid arthritis	4.13	11	24.79	25	1.8
RA 3	female	55	rheumatoid arthritis	5.26	106	481.40	18	1.5
RA 4	female	33	rheumatoid arthritis	6.26	13	>500	32	0.7
RA 5	female	39	rheumatoid arthritis	2.67	<10	<7	5	0
RA 6	female	18	rheumatoid arthritis	5.01	57	17.31	18	0.7
RA 7	female	42	rheumatoid arthritis	4.08	<10	<7	5	0.5
RA 8	female	47	rheumatoid arthritis	4.95	<10	<7	27	4.5
RA 9	female	51	rheumatoid arthritis	4.07	<10	<7	13	1.3
RA 10	female	50	rheumatoid arthritis	4.35	12	72.18	10	1.4
RA 11	female	55	rheumatoid arthritis	5.18	<10	<7	19	3.0
RA 12	female	42	rheumatoid arthritis	5.93	28	455	23	7.9
RA 13	male	55	rheumatoid arthritis	4.98	<10	<7	32	15.5
RA 14	female	23	rheumatoid arthritis	4.67	79	17.85	44	17.6
RA 15	male	51	rheumatoid arthritis	3.60	143	>500	14	1
HC 1	male	41	healthy control	-	<10	<7	6	0
HC 2	female	31	healthy control	-	<10	<7	5	0
HC 3	female	32	healthy control	-	<10	<7	21	13.7
HC 4	female	43	healthy control	-	<10	<7	19	0
HC 5	female	42	healthy control	-	<10	<7	8	6.2
HC 6	female	47	healthy control	-	<10	<7	15	1.7
HC 7	female	55	healthy control	-	11	<7	40	2.1
HC 8	female	46	healthy control	-	<10	<7	55	3.3
HC 9	female	34	healthy control	-	<10	<7	41	2.2
HC 10	male	57	healthy control	-	<10	<7	9	0.9
HC 11	female	48	healthy control	-	304	<7	4	0.7
HC 12	female	28	healthy control	-	<10	<7	10	0
HC 13	female	43	healthy control	-	<10	<7	9	2.7
HC 14	female	42	healthy control	-	<10	<7	9	0
HC 15	male	44	healthy control	-	<10	<7	33	4.2
HC 16	female	35	healthy control	-	<10	<7	8	0
HC 17	female	39	healthy control	-	<10	<7	22	10.1
HC 18	female	26	healthy control	-	<10	<7	10	0
HC 19	female	34	healthy control	-	<10	<7	4	3.3
HC 20	female	26	healthy control	-	<10	<7	2	0

**Table 2:** Primers used in qRT-PCR studies of bacterial DNA.

Primer Name	Sequence	Number of Nucleotides	Target Gene
Internal control, F	TTCTTAAGTCTGATGTGAAAAGC	22	16S rRNA
Internal control, R	TGGACTACCAGGGTATCTAATC	22	
*S. copri* genome-specific, F	TTTTGCTGTAGGAGGGGTTG	20	Glycosyl transferase, group 1 PREVCOP_06806
*S. copri* genome-specific, R	GGGCTGCATAAAGCAAAGAC	20	
*P. gingivalis* genome-specific, F	TCCACACCCGAAGCAGTAAC	20	hmuY gene
*P. gingivalis* genome-specific, R	TGCCACTTTCGCCACAATTG	20	

## Data Availability

All raw data will be made available upon request.

## Appendix A

**Table A1:** General and immunological characteristics of patients (HC—healthy controls, RA—patients with rheumatoid arthritis, ORD—patients with other rheumatic diseases).

Patients	Age	Sex	Disease Activity Score (DAS) 28	The Health Assessment Questionnaire (HAQ)	American College of Rheumatology (ACR) Joint Pain	ACR Joint Swelling	Total Score of Joint Pain	Total Score of Joint Swelling	Number of Painful Joints DAS28	Number of Swelling Joints DAS28	Rheumatoid Factor (RF)	Anti-Cyclic Citrullinated Peptide (Anti-CCP) [U/mL]	Neutrocytes [%]	Lymphocytes [%]	Monocytes [%]	Eosinophils [%]	Basophils [%]
ORD1	42	female	1.81	-	0	0	1	1	0	0	<10	-	45.6	42.3	9.3	2.5	0.3
ORD2	41	female	3.86	0.375	2	2	4	4	2	2	<10	<7	56.7	33.8	6.2	2.4	0.9
ORD3	22	female	4.56	0.500	5	0	5	0	18	0	<10	<7	62.5	26.0	10.1	1.3	0.1
ORD4	32	female	3.60	0.625	3	3	4	4	-	-	<10	<7	62.9	29.5	5.8	1.7	0.1
ORD5	34	female	0.64	0.250	1	0	2	1	0	0	17	<7	49.3	40.9	6.2	3.3	0.3
ORD6	38	female	2.10	0.250	3	2	4	3	3	1	<10	<7	51.4	38.0	8.5	1.9	0.2
ORD7	24	female	1.68	0.250	2	2	3	3	2	1	<10	<7	45.2	47.7	5.6	1.3	0.2
ORD8	46	female	2.91	-	3	0	4	1	5	0	<10	<7	53.1	33.1	10.5	2.9	0.4
ORD9	43	female	1.36	0	0	0	1	1	0	2	<10	<7	58.3	27.7	10.2	3.6	0.2
ORD10	23	female	4.17	1.000	5	5	9	9	26	0	96	<7	38.9	51.7	7.2	2.0	0.2
ORD11	20	female	3.38	0.750	3	0	6	3	10	0	23	<7	61.5	29.5	7.5	1.1	0.4
ORD12	46	female	2.04	0	-	-	-	-	0	0	<10	-	54.4	36.3	6.6	2.1	0.6
ORD13	52	female	3.53	0.875	-	-	-	-	2	1	<10	<7	46.6	41.1	8.5	3.5	0.3
ORD14	53	female	0	0	2	2	3	3	0	0	<10	<7	68.3	24.5	5.8	1.3	0.1
ORD15	56	female	-	-	0	0	1	1	-	-	<10	<7	41.9	46.3	8.7	2.7	0.4
ORD16	43	female	1.95	0	2	2	3	3	1	0	<10	-	-	-	-	-	-
ORD17	56	female	4.90	1.125	3	0	4	1	11	5	<10	<7	57.9	30.8	6.5	4.5	0.3
ORD18	44	female	2.02	0.625	2	2	3	3	0	3	<10	<7	52.4	35.4	7.3	3.4	1.5
ORD19	36	male	0.90	0	2	2	3	3	0	2	<10	<7	46.4	38.7	12.0	2.4	0.5
RA1	44	female	4.65	0.625	3	3	7	7	8	4	21	268.8	69.7	23.8	5.7	0.6	0.2
RA2	61	female	4.13	1.375	1	0	3	2	-	-	11	24.79	65.7	22.4	7.9	3.8	0.2
RA3	55	female	5.26	0.375	2	2	6	6	14	1	106	481.4	50.1	36.8	10.1	2.8	0.2
RA4	33	female	6.26	0.375	5	5	10	10	12	7	13	>500	71.0	20.9	6.1	1.6	0.4
RA5	39	female	2.67	0	3	0	3	0	4	0	<10	<7	54.4	30.7	8	6.7	0.2
RA6	18	female	5.01	1.375	2	5	6	9	10	10	57	17.31	45.4	43.0	8.2	3.1	0.3
RA7	42	female	4.08	0.375	3	3	4	4	10	1	<10	<7	59.8	29.1	8.4	2.5	0.2
RA8	47	female	4.95	0.875	2	3	2	3	4	6	<10	<7	66.7	22.0	8.6	2.5	0.2
RA9	51	female	4.07	0.750	3	2	4	4	7	3	<10	<7	67.4	20.2	8.8	3.1	0.5
RA10	50	female	4.35	1.625	2	2	5	5	3	1	12	72.18	71.4	21.8	4.9	1.6	0.3
RA11	55	female	5.18	0.750	5	5	6	6	8	8	<10	<7	56.5	31.2	7.2	4.3	0.8
RA12	42	female	5.93	2.000	5	5	10	10	12	8	28	455.0	63.6	23.5	8.5	4.1	0.3
RA13	55	male	4.98	0	2	2	4	4	-	-	<10	<7	67.9	21.6	6.7	3.4	0.4
RA14	23	female	4.67	0.625	3	3	8	8	1	10	79	17.85	71.2	17.2	9.3	2.1	0.2
RA15	51	male	3.60	0.875	3	3	7	7	4	0	143	>500	62.0	27.0	9.1	1.6	0.3
HC1	41	male	-	-	-	-	-	-	-	-	<10	<7	52.3	35.7	8.6	2.6	0.8
HC2	31	female	-	-	-	-	-	-	-	-	<10	<7	43.9	43.1	9.2	3.1	0.7
HC3	32	female	-	-	-	-	-	-	-	-	<10	<7	46.7	43.3	7.0	2.7	0.3
HC4	43	female	-	-	-	-	-	-	-	-	<10	<7	36.6	47.8	11.6	3.5	0.5
HC5	42	female	-	-	-	-	-	-	-	-	<10	<7	48.4	40.4	8.6	2.5	0.1
HC6	47	female	-	-	-	-	-	-	-	-	<10	<7	59.3	29.5	5.8	5.2	0.2
HC7	55	female	-	-	-	-	-	-	-	-	11	<7	56.5	35.6	6.4	1.2	0.3
HC8	46	female	-	-	-	-	-	-	-	-	<10	<7	55.5	32.2	8.4	3.7	0.2
HC9	34	female	-	-	-	-	-	-	-	-	<10	<7	60.4	30.5	6.3	2.1	0.7
HC10	57	male	-	-	-	-	-	-	-	-	<10	<7	51.4	34.3	11.9	2.0	0.4
HC11	48	female	-	-	-	-	-	-	-	-	304	<7	60.8	28.1	8.6	2.4	0.1
HC12	28	female	-	-	-	-	-	-	-	-	<10	<7	57.2	29.6	10.5	2.0	0.7
HC13	43	female	-	-	-	-	-	-	-	-	<10	<7	55.3	35.8	7.2	1.6	0.1
HC14	42	female	-	-	-	-	-	-	-	-	<10	<7	44.5	44.3	9.8	0.9	0.5
HC15	44	male	-	-	-	-	-	-	-	-	<10	<7	65.9	25.5	7.3	1.2	0.1
HC16	35	female	-	-	-	-	-	-	-	-	<10	<7	57.6	30.0	11.4	0.8	0.2
HC17	39	female	-	-	-	-	-	-	-	-	<10	<7	57.2	30.1	8.8	3.4	0.5
HC18	26	female	-	-	-	-	-	-	-	-	<10	<7	41.8	44.8	9.7	3.0	0.7
HC19	34	female	-	-	-	-	-	-	-	-	<10	<7	38.3	47.6	11.0	2.9	0.2
HC20	26	female	-	-	-	-	-	-	-	-	<10	<7	54.9	31.3	11.0	2.7	0.1

## Appendix B

**Table A2:** Patients’ biochemical features (HC, RA, and ORD).

Patients	Hemoglobin [g/dL]	Hematocrit [%]	Mean Corpuscular Volume (MCV) [fL]	Mean Corpuscular Hemoglobin (MCH) [pg/cell]	Mean Corpuscular Hemoglobin Concentration (MCHC) [g/dL]	Red Cell Distribution Width (RDW) [%]	Platelet Count Test (PLT) × 10^9^/L	Mean Platelet Volume (MPV) [fL]	Erythrocyte Sedimentation Rate (ESR) [mm/hr]	Glucose [mg/dL]	Creatinine [mg/dL]	Glomerular Filtration Rate (GFR) [mL/min/1.73m^2^]	Uric Acid [mg/dL]	Albumin [g/dL]	C-Reactive Protein (CRP) [mg/dL]	Alanine Aminotransferase (ALAT) [U/L]
ORD1	11.9	37.1	81.2	26.0	32.1	14.1	266	10.0	6	92	0.85	>60	4.3	46.5	0	11
ORD2	14.0	41.9	86.0	28.7	33.4	13.1	478	9.8	23	97	0.65	>60	4.7	49.0	5.9	19
ORD3	13.4	40.7	91.9	30.2	32.9	13.7	261	10.5	26	88	0.82	>60	6.1	41.3	0.4	10
ORD4	14.2	42.2	84.7	28.5	33.6	12.9	266	11.2	18	109	0.58	>60	5.3	46.3	1.3	22
ORD5	12.3	36.3	87.5	29.6	33.9	12.6	226	12.1	2	91	0.82	>60	3.6	44.0	0.3	12
ORD6	14.1	41.0	87.8	30.2	34.4	12.4	209	11.7	2	91	0.65	>60	3.8	46.0	0.3	15
ORD7	14.5	42.0	82.7	28.5	34.5	12.1	200	11.6	2	91	0.80	>60	5.1	50.6	0	12
ORD8	12.2	35.5	87.7	30.1	34.4	13.1	334	10.4	4	85	0.67	>60	3.7	47.4	0	11
ORD9	13.0	38.8	94.6	31.7	33.5	13.5	318	9.6	4	85	0.87	>60	4.0	47.3	0.5	10
ORD10	14.4	41.9	88.4	30.4	34.4	12.4	238	11.7	3	79	0.86	>60	5.3	51.7	0	16
ORD11	14.1	41.4	88.5	30.1	34.1	12.9	262	11.1	2	107	0.80	>60	3.7	50.0	0	16
ORD12	12.5	38.5	80.5	26.2	32.5	15.4	314	11.8	17	100	0.85	>60	3.8	48.7	1.3	16
ORD13	15.5	45.1	95.6	32.8	34.4	13.3	284	10.9	15	99	0.71	>60	3.6	51.6	0	15
ORD14	13.9	42	89.9	29.8	33.1	13.2	334	10.0	26	93	0.71	>60	3.2	46.8	0.8	11
ORD15	14.2	41.9	86.4	29.3	33.9	12.7	229	10.5	9	93	0.84	>60	4.5	45.5	0.5	14
ORD17	13.9	39.6	89.6	31.4	35.1	12.1	202	11.9	8	86	0.76	>60	4.9	49.1	2.8	28
ORD18	12.2	37.3	82.9	27.1	32.7	15.4	290	11.5	7	88	0.88	>60	-	-	0	13
ORD19	16.8	47.6	88.5	31.2	35.3	12.9	254	10.1	2	82	0.80	>60	5.5	48.2	0.7	29
RA1	12.9	38.6	89.6	29.9	33.4	13.5	372	10.4	8	83	0.64	>60	3.1	46.1	1.3	31
RA2	13.6	40.4	87.1	29.3	33.7	13.1	271	10.9	25	98	0.97	59	5.3	44.5	1.8	19
RA3	12.9	37	82.6	28.8	34.9	13.5	214	12.4	18	88	0.75	>60	4.3	48.6	1.5	14
RA4	12.4	36.4	86.7	29.5	34.1	13.4	245	10.4	32	1	0.68	>60	3.3	49.3	0.7	18
RA5	12.8	36.7	85.2	29.7	34.9	17.6	275	10.7	5	95	0.79	>60	3.5	46.9	0	18
RA6	13.6	40.5	85.6	28.8	33.6	13.2	327	11.9	18	91	0.62	>60	-	40.4	0.7	11
RA7	12.6	38.7	86.4	28.1	32.6	12.9	224	11.8	5	87	0.65	>60	3.6	44.4	0.5	12
RA8	12.7	37.9	83.1	27.9	33.5	13.0	303	10.3	27	96	0.61	>60	2.2	45.0	4.5	14
RA9	14.1	42	90.3	30.3	33.6	13.8	318	11.6	13	1	0.71	>60	5.2	45.9	1.3	26
RA10	13.4	38.4	89.5	31.2	34.9	13.0	174	10.1	10	91	0.81	>60	4.6	46.3	1.4	32
RA11	12.9	40.7	87	27.6	31.7	12.9	225	10.5	19	112	0.60	>60	6.1	45.2	3.0	28
RA12	13.6	40.8	81.4	27.1	33.3	14.7	346	10.6	23	93	0.82	>60	4.5	41.2	7.9	11
RA13	12.4	38.8	77.8	24.8	32.0	23.2	330	10.2	32	96	0.89	>60	3.7	41.3	15.5	23
RA14	10.9	34.5	79.7	25.2	31.6	14.1	501	10.2	44	88	0.61	>60	3.9	41.7	17.6	8
RA15	14.6	43.4	91.9	30.9	33.6	13.1	259	10.2	14	139	0.82	>60	4.2	42.6	1.0	12
HC1	15.1	45.7	79.5	26.3	33.0	13.9	284	10.4	6	85	0.91	>60	5.7	47.0	0	26
HC2	13.8	41.1	91.7	30.8	33.6	13.6	296	9.7	5	89	0.83	>60	5.0	0.83	0	11
HC3	12.8	37.2	89.2	30.7	34.4	12.2	296	9.7	21	84	0.84	>60	3.6	42.9	13.7	15
HC4	11.5	34	82.3	27.8	33.8	14.3	309	10.2	19	88	0.71	>60	3.4	46.0	0	15
HC5	14.2	41.1	87.4	30.2	34.5	12.5	260	10.9	8	89	0.96	>60	3.2	42.7	6.2	10
HC6	12.7	37.9	91.3	30.6	33.5	13.2	296	10.4	15	95	0.82	>60	3.1	43.6	1.7	12
HC7	12.9	37.8	90.9	31	34.1	12.4	280	11.2	40	87	0.76	>60	4.6	42.4	2.1	16
HC8	10.7	32.7	74.3	24.3	32.7	16.4	360	9.7	55	84	0.73	>60	3.9	0.7	3.3	16
HC9	12.4	37.6	76.7	25.3	33.0	15.7	344	10.9	41	101	0.88	>60	6.8	45.1	2.2	21
HC10	15.9	45.6	86.7	30.2	34.9	13.9	151	11.4	9	107	1.20	>60	6.4	44.3	0.9	20
HC11	14.4	42.1	90.7	32.2	34.7	12.3	214	11.4	4	82	0.75	>60	4.0	43.8	0.7	16
HC12	14.3	40.4	82.1	29.1	35.4	13.6	170	12.6	10	96	1.02	>60	4.9	46.4	0	7
HC13	15	43	83.5	29.1	34.9	11.8	342	10.6	9	87	0.79	>60	3.4	46.3	2.7	20
HC14	13.1	38.7	93	31.5	33.9	13.7	338	11.5	9	89	0.65	>60	3.4	44.0	0	14
HC15	13.4	39.1	85	29.1	34.3	13.5	235	12.5	33	88	0.75	>60	5.0	0.8	4.2	17
HC16	12.5	37.7	92	30.5	33.2	14.0	285	10.4	8	83	0.75	>60	3.3	44.5	0	8
HC17	14	41.6	78.9	26.6	33.7	13.3	275	9.5	22	84	0.77	>60	5.7	42.1	10.1	14
HC18	13.1	38.2	86.8	29.8	34.3	12.9	229	11.8	10	83	0.79	>60	3.9	48.0	0	11
HC19	13	38.2	84.5	28.8	34.0	13.3	259	12.1	4	76	0.77	>60	3.8	41.2	3.3	17
HC20	13.4	39.1	92.9	31.8	34.3	12.5	244	12.3	2	87	0.67	>60	5.5	47.0	0	17
